# Efficacy of Naprapathy in Brachial Plexus Injury: Protocol for a Randomized Clinical Trial

**DOI:** 10.2196/46054

**Published:** 2023-05-29

**Authors:** Bin Xiao, Lishu Zhao, Yong Huang, Anqi Ma, Baoshun Pei, Zhengyu Li, Fei Gu

**Affiliations:** 1 Shanghai University of Traditional Chinese Medicine Shanghai China; 2 Shanghai Literature and Art Hospital Shanghai China; 3 Yueyang Hospital affiliated with Shanghai University of Traditional Chinese Medicine Shanghai China; 4 Shanghai Putuo Hospital affiliated with Shanghai University of Traditional Chinese Medicine Shanghai China

**Keywords:** naprapathy, brachial plexus injury, traditional Chinese medicine, study protocol, clinical rehabilitation, chronic pain, physical therapy, randomized controlled trial, neuromusculoskeletal, acupuncture, moxibustion, Tuina, TCM, pain, edema, blood circulation, brachial plexus, spinal cord, limb, electromyography, EMG

## Abstract

**Background:**

Clinical rehabilitation for brachial plexus injury is difficult in terms of chronic pain and dysfunction. Physiotherapy is considered a routine intervention for rehabilitation. Common physical therapy may require a variety of instruments. One approach that does not need instruments, but belongs to the field of complementary and alternative medicine, is naprapathy. Naprapathy, also called Tuina in China, has been applied in rehabilitation after brachial plexus injury for a long time. Naprapathy can relieve chronic neuropathic pain, promote local blood circulation, and improve body edema. Naprapathy can passively help improve motor functions in patients with peripheral nerve injury. However, the efficacy of naprapathy in improving rehabilitation after brachial plexus injury is unclear.

**Objective:**

This study aims to evaluate the additional value of naprapathy when combined with conventional physical therapy for the treatment of brachial plexus injury.

**Methods:**

This will be a single-center randomized controlled trial. A total of 116 eligible patients with brachial plexus injury will be randomly divided into an experimental group (naprapathy plus physical therapy group) or a control group (physical therapy group). The participants will be followed up for 4 weeks of treatment. Observation outcomes will include the visual analog scale score, upper limb index, electromyography findings, and adverse reactions, among others. The measuring points for outcomes will be the baseline and the completion of treatment. In addition, a quality control group independent from the research team will be set up to control the quality of the trial. Finally, the data will be analyzed using SPSS software (version 21.0; IBM Corp).

**Results:**

The study is recruiting participants. The first participant was enrolled in September 2021. As of January 2023, a total of 100 participants have been enrolled. The trial is expected to be completed by September 2023. The study protocol was approved by the Ethics Review Committee of Yue Yang Hospital affiliated with the Shanghai University of Traditional Chinese Medicine (2021-012).

**Conclusions:**

One limitation of this trial is that we will be unable to achieve strict double-blinding because of the features of naprapathy. The trial aims to contribute reliable evidence for decision-making in naprapathy for treating brachial plexus injury.

**Trial Registration:**

Chinese Clinical Trial Registry ChiCTR2100043515; http://www.chictr.org.cn/showproj.aspx?proj=122154

**International Registered Report Identifier (IRRID):**

DERR1-10.2196/46054

## Introduction

Brachial plexus injury (BPI) is one of the most challenging problems in modern medicine because of its high disability rate, surgical difficulty, and complex sequelae [1]. It is mostly caused by injuries such as a motorcycle accident, birth injury, bruising, puncture injury, fracture, dislocation, and lung apex tumor resection [2-5]. Sensory impairment or muscle paralysis and atrophy, which cause partial or complete upper limb motor and sensory dysfunction, may place a great burden on the patient's life and mental health [6,7]. With the development of microsurgical technology in recent decades [8], great progress has been made in the treatment of BPI, especially in terms of surgical treatments [9,10], allowing good conditions for the functional recovery of patients' limbs to be provided. There are also some rehabilitation methods, such as medium-frequency electrical stimulation, infrared irradiation, ultrasound therapy, and so forth, but the effects are not satisfactory [11,12].

The ability of an injured nerve to regenerate is closely related to the repair time, with 1 to 3 months after injury being considered the “golden period” for nerve repair [13]. However, with a delayed diagnosis, the prognosis is poor. Surgical repair is only performed in preparation for the treatment of BPI; rehabilitation is the beginning of treatment and is necessary after surgery [14]. Due to differences in the condition of patients with BPI, physical therapy should be used to relieve pain, eliminate swelling, prevent scar adhesion, and joint contracture; promote nerve regeneration; and prevent muscle atrophy and joint stiffness [15,16].

At present, physical rehabilitation therapies commonly used after BPI, such as neuromyoelectric stimulation, intermediate frequency electrotherapy, and hand function training [17-19], have certain effects on upper limb function, but the effect of rehabilitation on hand function remains limited.

Naprapathy (also called Tuina therapy in traditional Chinese medicine) [20] consists of physical stimuli acting on the human body through manipulation to cause physiological responses in tissue fibers. Through the regulation of nerve reflexes and body fluids, therapeutic effects on damaged areas can be achieved. Naprapathy can promote capillary dilation, increase blood circulation, and improve muscle blood circulation such that the condition of damaged tissue can be improved and the tissue will be repaired [21]. Swelling and cramping can also be reduced or alleviated. Our previous study showed that naprapathy can improve the pain threshold in rats with neuropathic pain caused by BPI, increase the expression of β-endorphin and gamma-aminobutyric acid in the reward circuit in the brain [22], and relieve chronic neuropathic pain in rats. These findings may represent one of the neurological mechanisms of naprapathy. Naprapathy not only exerts an analgesic effect but also provides a certain comfort for patients. Patients with BPI are also willing to undergo naprapathy in the clinic. However, there is a lack of clinical research on naprapathy in the treatment of BPI. Therefore, we designed this randomized controlled trial (RCT) to evaluate the additional effects of naprapathy when combined with conventional physical therapy on pain and hand motor function in patients with BPI.

## Methods

### Study Design

**Figure 1:**
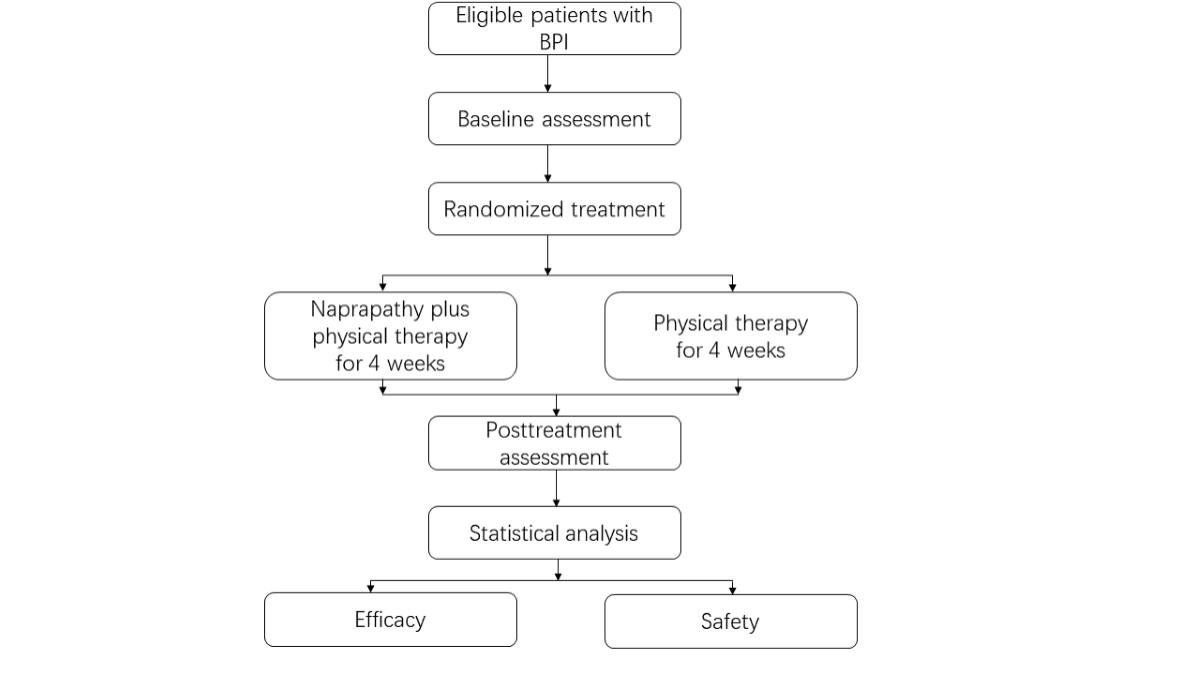
Study flowchart. BPI: brachial plexus injury.

This clinical trial will be an open-label RCT designed to compare naprapathy plus physical therapy and physical therapy in treating BPI. This protocol has been registered on the Chinese Clinical Trial Registry (2100043515) and will be performed in accordance with the Declaration of Helsinki. The study flowchart is shown in Figure 1.

### Study Setting of Recruitment

Recruitment will be carried out at Yue Yang Hospital affiliated with the Shanghai University of Traditional Chinese Medicine. A total of 116 participants with BPI will be enrolled. Recruitment will be carried out through social platforms and outpatient departments. The study team will educate physicians about the study, focusing on those who do the surgery for patients with BPI. If the patients meet the inclusion criteria and are willing to participate in the trial, they will be asked to provide written informed consent. Then, they will be randomized into the naprapathy plus physical therapy group (group A) or the physical therapy group (group B) by LZ and BP.

### Ethics Approval

The study protocol was approved by the ethics review committee of Yue Yang Hospital affiliated with the Shanghai University of Traditional Chinese Medicine (2021-012). Any changes including eligibility criteria, outcomes, and so forth should be reported to the Ethics Review Committee. The ethics committee of Yueyang Hospital affiliated with the Shanghai University of Traditional Chinese Medicine will audit the whole procedure during the clinical trial conducted independently from investigators and the funder. The participants signed the informed consent and agreed to the primary and secondary analyses of their data without additional consent. All the study data are deidentified. This trial has also been registered in the Chinese Clinical Trial Registry (2100043515). The results of the preliminary study will be published in a professional journal.

### Patient and Public Involvement

Patients and the public are involved in the design of the study. They reviewed the study proposal and attended the design meetings. Patients and the public will be included in all research communications.

The inclusion criteria are as follows: (1) aged 18 to 70 years; (2) BPI diagnosed by history, physical examination, electromyography, or surgical exploration more than 2 weeks after nerve surgery; (3) agreement to provide written informed consent.

The exclusion criteria are as follows: (1) concurrent damage to major organs, such as the heart, kidneys, liver, and so forth; (2) severe cardiovascular or cerebrovascular disease, liver or kidney dysfunction, metabolic disease, or mental illness; (3) chronic illness, physical discomfort, frailty, severe neurosis, or pregnancy; (4) primary brachial plexus pain or brachial plexus neuritis; (5) brachial plexus pain due to cervical spondylosis, tennis elbow, carpal tunnel syndrome, frozen shoulder, biceps tenosynovitis, thoracic outlet syndrome, or other secondary causes.

The participants will be withdrawn if they have the following: (1) missing or incomplete patient treatment records, which would affect the final data analysis; (2) treatment with other methods at the same time; (3) any severe adverse reactions or other emergent symptoms during the treatment (such as aggravation of disease or severe depression and the need for special treatment).

### Randomization

In this clinical trial, a total of 116 participants with BPI will be randomly assigned to either group A (naprapathy plus physical therapy) or group B (physical therapy) at a 1:1 ratio. The random sequence will be generated using SAS software (version 9.3; SAS Institute, Inc). The coauthors randomizing the participants are not linked to any statistical work.

### Interventions

The treatment plan of this trial is based on naprapathy in clinical rehabilitation for the treatment of BPI. Participants in group A will receive naprapathy in addition to physical therapy, while participants in group B will receive physical therapy only. Treatment in both groups will last 4 weeks. The interventions in this trial involve rigorous naprapathy and physical therapy schedules. To ensure the compliance of naprapathy practitioners and physical therapists with their schedules, they will be asked to undergo pretrial training, after which they will be required to pass an entrance examination to participate in this trial. All included naprapathy practitioners and physical therapists will have at least 5 years of practical experience in clinical rehabilitation.

### Physical Therapy

All participants will receive physical therapy, such as neuromyoelectric stimulation by a transcutaneous electrical nerve stimulator (TENS 21, Homer Ion), medium-frequency electrotherapy by a medium-frequency electrical stimulator (ES-521, Ito), and continuous passive motion by an upper limb continuous passive motion joint rehabilitation device (2029, DJO), once each day for 5 continuous days in a week. Each therapy session will last for 40 minutes and will be performed 5 times a week (from Monday to Friday) for a total of 20 sessions in 4 weeks.

### Naprapathy

**Figure 2:**
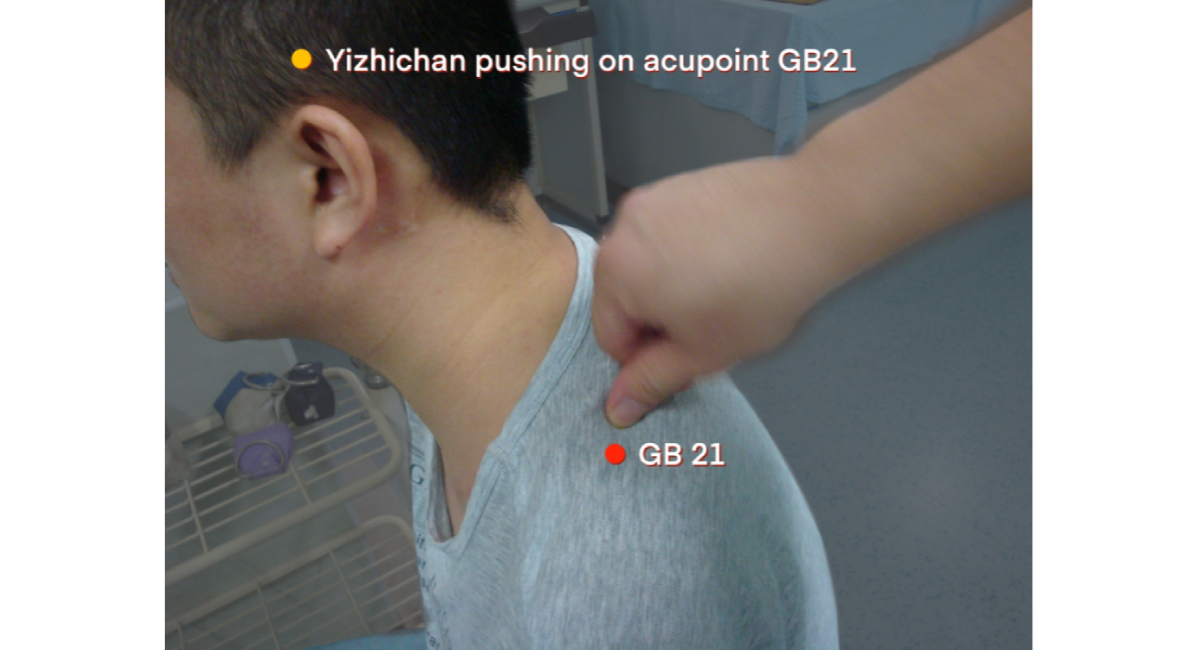
Yizhichan pushing manipulation.

**Figure 3:**
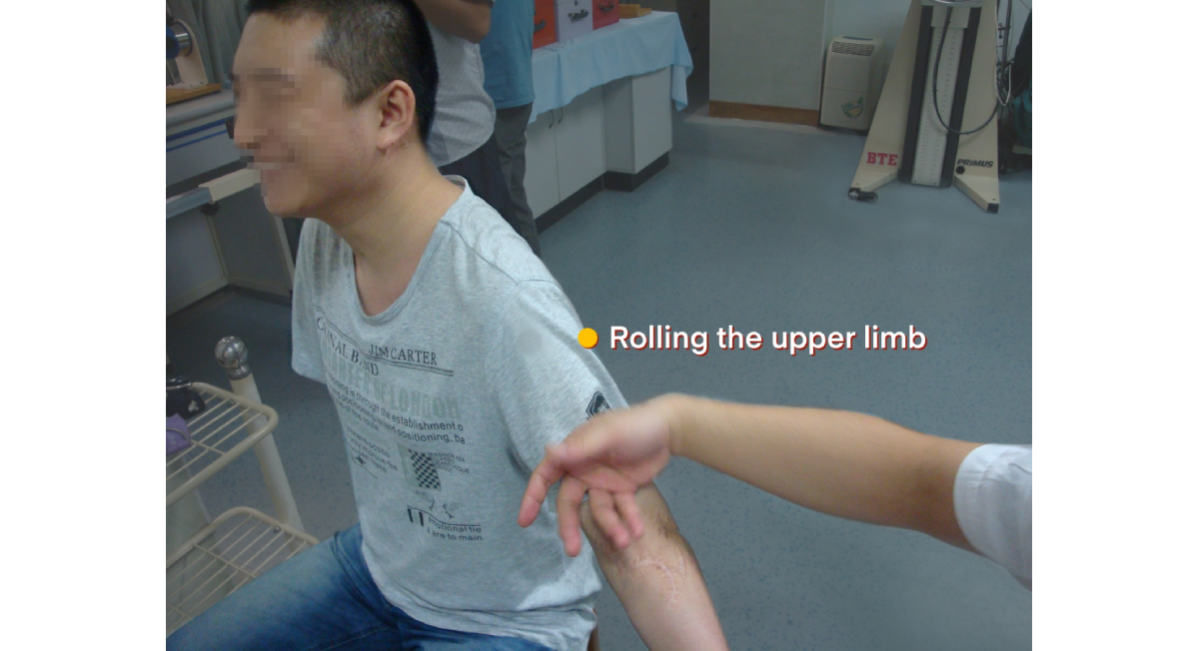
Rolling manipulation.

**Figure 4:**
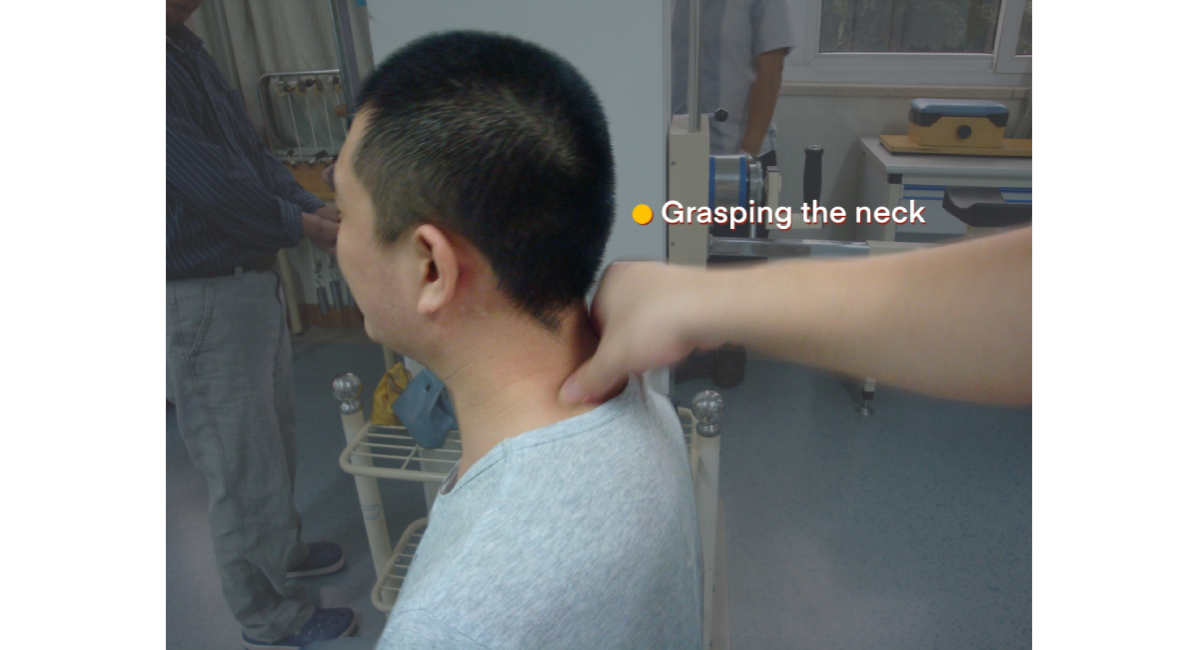
Grasping manipulation.

**Figure 5:**
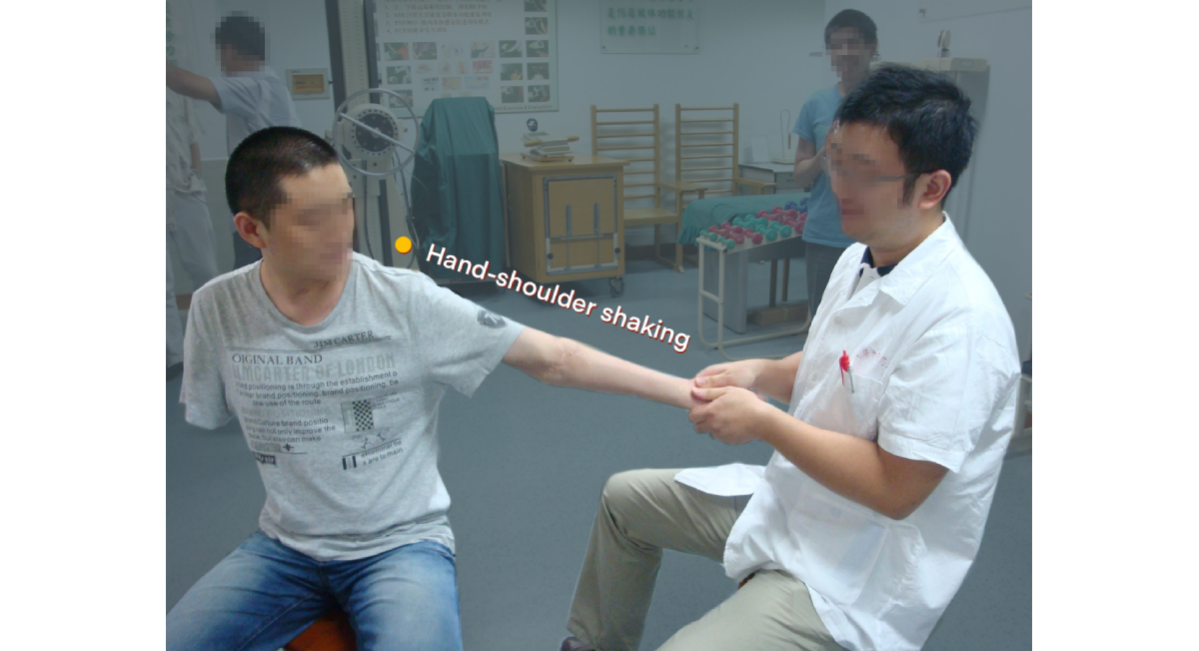
Shaking manipulation.

For patients with total BPI, the patient will take a sitting position. The naprapathy practitioner will stand on the affected side and first apply Yizhichan (one-finger Zen) (Figure 2) pushing manipulation and press-kneading manipulation at Fengchi (GB 20, affected side), cervical Jiaji points (EX-B2, affected side), Jianjing (GB 21, affected side), Jianyu (LI 15, affected side), Quchi (LI 11, affected side), Waiguan (SJ 5, affected side), and Hegu (LI 4, affected side). Then, the practitioner will apply rolling manipulation (Figure 3) from the affected shoulder to the upper limb and grasping manipulation (Figure 4) on the neck, shoulder, and upper limb. Finally, the hand-shoulder shaking (Figure 5) method will be applied to help soothe the joints. Within the allowable safety range of the affected limbs, the joints of the upper limbs will be passively moved.

For patients with upper trunk BPI, the naprapathy practitioner will apply Yizhichan pushing and press-kneading manipulations at Fengchi (GB 20, affected side), Yunmen (LU 2, affected side), Quchi (LI 11, affected side), Shousanli (LI 10, affected side), Hegu (LI 4, affected side), Yangxi (LI 5, affected side), Sanjian (LI 3, affected side), Yuji (LU 10, affected side), and Laogong (PC 8, affected side). The other methods will be the same as above.

For patients with lower trunk BPI, the naprapathy practitioner will apply Yizhichan pushing and press-kneading manipulations at Tianzong (SI 11, affected side), Jianzhen (SI 9, affected side), Waiguan (SJ 5, affected side), Yangchi (SJ 4, affected side), Jianwaishu (SI 14, affected side), Xiaohai (SI 8, affected side), Yanglao (SI 6, affected side), Yanggu (SI 5, affected side), and Houxi (SI 3, affected side). The other methods will be the same as above.

The duration of each naprapathy session will be 20 minutes and will be performed 5 times a week (every Monday to Friday) for a total of 20 sessions in 4 weeks. If there are any adverse events, the treatment will be suspended, and the investigator will decide whether to terminate the treatment.

**Figure 6:**
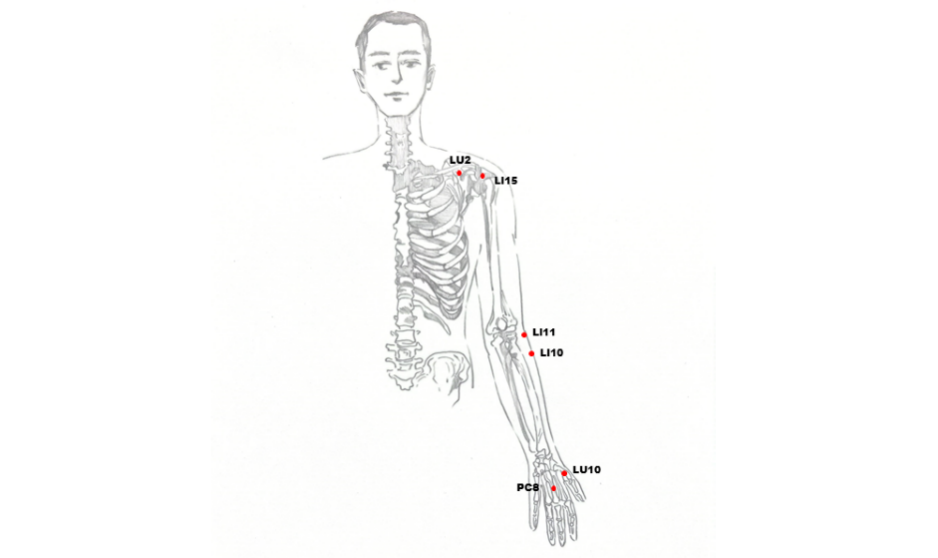
Illustration of acupoints (front).

**Figure 7:**
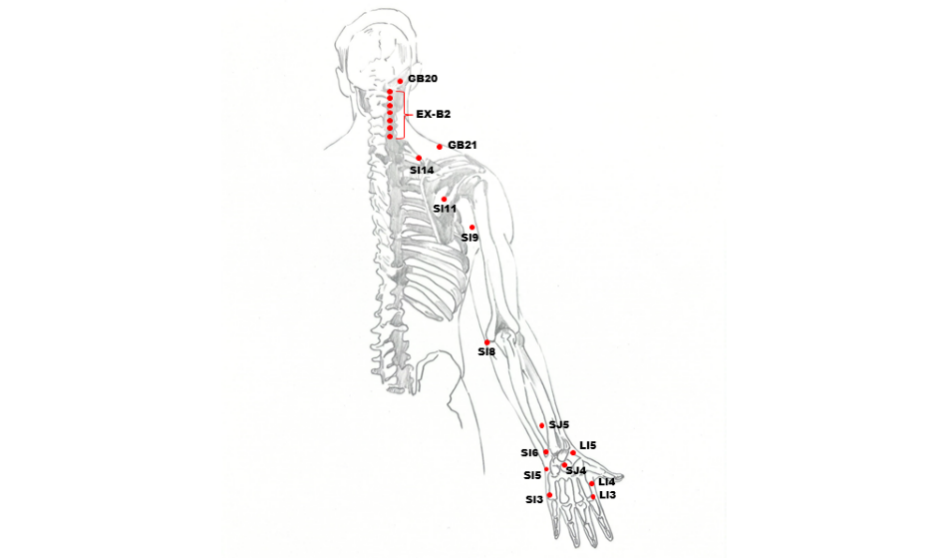
Illustration of acupoints (back).

All the acupoints used in this study are shown in Figures 6 and 7.

### Outcomes

#### Primary Outcome Indicator

The primary outcome measure will be the change in visual analog scale score relative to baseline at 4 weeks.

#### Secondary Outcome Indicators

The secondary outcomes will include the upper limb motor function score according to the Brunnstrom functional assessment and electromyography findings.

### Time Points of Outcome Measurement

Outcomes will be evaluated at 2 time points: baseline and at the completion of treatment. An overview of the outcomes measured at the different time points is shown in Table 1.

**Table 1:** Case data collection timetable.

Time point	Baseline	Posttreatment
Written informed consent	✓	
Concomitant treatment	✓	
Disease history	✓	
History of medication	✓	
Brunnstrom functional assessment	✓	✓
VAS^a^	✓	✓
EMG^b^ examination	✓	✓
Participant self-evaluation		✓
Adverse events		✓
Safety analysis		✓

^a^VAS: visual analog scale.

^b^EMG: electromyography.

### Assessment of Safety and Adverse Events

According to clinical experience and the literature, naprapathy may cause several kinds of adverse events, such as subcutaneous hematoma, edema, skin bruising, local pain, and fainting. Among these adverse events, subcutaneous hematoma and edema are the most common. Naprapathy-related safety and adverse events will be carefully recorded using case report forms. Adverse events should be reported to the ethics committee of Yueyang Hospital affiliated with the Shanghai University of Traditional Chinese Medicine. The Ethics Committee has the right to stop the intervention of the participant.

### Data Collection and Management

All our measures will be free of charge to ensure complete participation in the follow-up evaluation. Case report forms will be completed in paper form and stored by the Shanghai University of Traditional Chinese Medicine. Data will be collected according to Table 1 by investigators, and personal information about the enrolled participants will be kept in Yueyang Hospital in order to protect confidentiality in the whole trial. The ethics committee of Yueyang Hospital affiliated with the Shanghai University of Traditional Chinese Medicine will audit the trial every 12 months and will establish a data monitoring committee, which is independent from the funder and has no competing interests. The data will be stored in the clinical trial public management platform and ready for public inquiry after the trial. A quality control group independent from the research team will be set up to control the quality of this study.

### Sample Size

Our previous study indicated that 58.5% of patients with BPI showed improvements in upper extremity function and muscle strength [23]. In this clinical trial, the rate of upper extremity function recovery is expected to be 75% in the naprapathy group and 55% in the physical therapy group. With α=.05 and β=.01, a total of 52 participants will be needed, as calculated using SPSS software (version 21.0; IBM Corp). According to the previous study, a 10% withdrawal rate will also be considered; therefore, each group will include at least 58 participants.

### Statistical Analysis

All data will be analyzed by researchers using SPSS (version 21.0) software. Data will be expressed as the mean (SD) or median (IQR). First, the data will be tested for normality and homogeneity of variance; if the data meet the requirements for a normal distribution, the homogeneity of variance will be analyzed by the *F* test. In the case of heterogeneity, the corrected *t* test will be used. If the requirements for normality and homogeneity of variance are not satisfied, the rank-sum test will be used. If the sphericity test yields a *P* value less than .05, indicating that the assumption of sphericity is violated, we will perform multivariate analysis. A statistically significant result (*P*<.05) for the time factor will indicate that the measurements show a trend of changing over time. A statistically significant (*P*<.05) interaction between time and group will indicate that in addition to the effect of time, there are differences between the groups. If a *P* value is less than .05, the difference will be considered statistically significant, indicating a difference in the results between the two groups. If a *P* value is greater than .05, then the difference will not be considered statistically significant, indicating no difference in the results between the two groups. Cohen *d* (sample size) will also be used to compare differences between groups, and the generalized linear model will be applied to explore differences in outcomes between groups.

For safety analysis, adverse event data are generally expressed as the number of events and the percentage of cases involved in the adverse event.

## Results

This study was funded by The National Natural Science Foundation of China and the Shanghai Health Commission. Yue Yang Hospital affiliated with the Shanghai University of Traditional Chinese Medicine approved the study protocol in February 2021. The first participant was enrolled in September 2021. As of January 2023, a total of 100 participants have been enrolled. The trial is expected to be completed by September 2023.

## Discussion

Due to methodological design problems, the quality of currently available clinical studies on naprapathy for BPI is relatively low. Therefore, this study is designed as an RCT to evaluate the additional effects of naprapathy when combined with physical therapy on BPI in terms of pain and upper extremity motor function.

Additionally, this single-center trial will provide reliable data for our future multicenter RCTs. Participants will receive 5 treatments a week, which is the most common frequency for external traditional Chinese medicine treatments for rehabilitation after BPI.

One limitation of this trial is that we will be unable to achieve strict double-blinding because of the features of naprapathy. Other limitations include manpower, material resources and funds, and short follow-up time, and it is expected that the future study will extend the follow-up time. Naprapathy relies on the intervention of the naprapathy practitioner, and future trials could be designed with a sham naprapathy placebo group.

This study aims to provide reliable evidence for naprapathy in the treatment of BPI and will compensate for the lack of related research on clinical efficacy.

## Abbreviations

BPI: brachial plexus injury
RCT: randomized controlled trial

## References

[1] Clapp MA, Bsat J, Little SE, Zera CA, Smith NA, Robinson JN (2016). Relationship between parity and brachial plexus injuries. J Perinatol.

[2] Kaiser R, Waldauf P, Ullas G, Krajcová A (2020). Epidemiology, etiology, and types of severe adult brachial plexus injuries requiring surgical repair: systematic review and meta-analysis. Neurosurg Rev.

[3] Abzug JM, Mehlman CT, Ying J (2019). Assessment of current epidemiology and risk factors surrounding brachial plexus birth palsy. J Hand Surg Am.

[4] Ozaras N, Ergin O, Eroglu Demir S, Sariyildiz MA (2014). Brachial plexus injury complicating the labor: mother is the victim at this time. J Matern Fetal Neonatal Med.

[5] Chang C-Y, Wu Y-T, Chen L-C, Chan R-C, Chang S-T, Chiang S-L (2015). Massage-induced brachial plexus injury. Phys Ther.

[6] Belviso I, Palermi S, Sacco AM, Romano V, Corrado B, Zappia M, Sirico F (2020). Brachial plexus injuries in sport medicine: clinical evaluation, diagnostic approaches, treatment options, and rehabilitative interventions. J Funct Morphol Kinesiol.

[7] Lovaglio AC, Socolovsky M, Di Masi G, Bonilla G (2019). Treatment of neuropathic pain after peripheral nerve and brachial plexus traumatic injury. Neurol India.

[8] Ayhan E, Soldado F, Fontecha CG, Bertelli JA, Leblebicioglu G (2020). Elbow flexion reconstruction with nerve transfer or grafting in patients with brachial plexus injuries: a systematic review and comparison study. Microsurgery.

[9] Bjorklund KA, West JM, Nopkhun W, Moore AM (2021). Surgical innovations to restore function in pediatric peripheral nerve conditions. Pediatrics.

[10] Bertelli JA, Ghizoni MF (2022). Reconstruction of C5-C8 (T1 hand) brachial plexus paralysis in a series of 52 patients. J Hand Surg Am.

[11] Smania N, Berto G, La Marchina E, Melotti C, Midiri A, Roncari L, Zenorini A, Ianes P, Picelli A, Waldner A, Faccioli S, Gandolfi M (2012). Rehabilitation of brachial plexus injuries in adults and children. Eur J Phys Rehabil Med.

[12] Rich JA, Newell A, Williams T (2019). Traumatic brachial plexus injury rehabilitation using neuromuscular electrical muscle stimulation in a polytrauma patient. BMJ Case Rep.

[13] Lee JA, Smith BT, Egro FM, Stanger M, Koster W, Grunwaldt LJ (2021). Timing of nerve recovery after nerve grafting in obstetrical brachial plexus palsy patients with isolated upper trunk neuromas. Ann Plast Surg.

[14] Brito S, White J, Hill B, Thomacos N (2022). Effective long-term management of brachial plexus injury following surgery: what is needed from hand therapists' perspectives. J Hand Ther.

[15] Huang H, Chen S, Wu L, Dou S, Chen Q, Li Y, Xiao Z, Wu H, Chen S (2021). Therapeutic strategies for brachial plexus injury. Folia Neuropathol.

[16] de Assis EDB, Martins WKN, de Carvalho CD, Ferreira CM, Gomes R, de Almeida Rodrigues ET, Meira UM, de Holanda LJ, Lindquist AR, Morya E, Mendes CKTT, de Assis TCG, de Oliveira EA, Andrade SM (2022). Effects of rTMS and tDCS on neuropathic pain after brachial plexus injury: a randomized placebo-controlled pilot study. Sci Rep.

[17] Stonner MM, Mackinnon SE, Kaskutas V (2021). Predictors of functional outcome after peripheral nerve injury and compression. J Hand Ther.

[18] Hsieh Y-L, Yang N-P, Chen S-F, Lu Y-L, Yang C-C (2022). Early intervention of cold-water swimming on functional recovery and spinal pain modulation following brachial plexus avulsion in rats. Int J Mol Sci.

[19] de Santana Chagas AC, Wanderley D, de Oliveira Ferro JK, de Moraes AA, de Souza FHM, da Silva Tenório A, de Oliveira DA (2022). Physical therapeutic treatment for traumatic brachial plexus injury in adults: a scoping review. PM R.

[20] Xiao B, Ma A, Li Z, Zhang S, Xu X, Zhou J, Li W, Zhang J, Yao F (2020). Naprapathy attenuates neuropathic pain after brachial plexus injury. Ann Palliat Med.

[21] Lilje S, Eklund A, Wykman A, Sundberg T, Skillgate E (2021). Naprapathy versus orthopaedic standard care for common musculoskeletal disorders: an 8-year follow-up of a pragmatic randomized controlled trial in Sweden. Chiropr Man Therap.

[22] Xiao B, Li Z, Yu Z-Y, Zhang J, Yan J-J, Liu X (2018). Experimental study on the effect of an-pressing and rou-kneading huantiao (GB 30) on certain brain nuclei of pleasure circuits in rats with chronic neuralgia. J Acupunct Tuina Sci.

[23] Yue XY, Zheng-Yu LI, Xiao B, Zhou JM, Wang WL (2016). The efficacy of tuina therapy in treating patients with total brachial plexus injury. J Guiyang Coll Tradit Chin Med.

